# Expression of Amine Oxidase Proteins in Adrenal Cortical Neoplasm and Pheochromocytoma

**DOI:** 10.3390/biomedicines11071896

**Published:** 2023-07-04

**Authors:** Eun Kyung Kim, Ja Seung Koo

**Affiliations:** 1 Department of Pathology, National Health Insurance Service Ilsan Hospital, Goyang 10444, Republic of Korea; 2 Department of Pathology, Yonsei University College of Medicine, Seoul 03722, Republic of Korea

**Keywords:** adrenal gland tumor, adrenal cortical tumor, pheochromocytoma, amine oxidase protein

## Abstract

We delved into the expression of amine oxidase family proteins and their potential significance in adrenal gland neoplasm. Tissue microarrays were prepared for 132 cases of adrenal cortical neoplasm (ACN) consisting of 115 cases of adrenal cortical adenoma (ACA), 17 cases of adrenal cortical carcinoma (ACC), and 163 cases of pheochromocytoma (PCC). Immunohistochemical stainings for MAOA, MAOB, LOX, and AOC3 were performed to evaluate the H-scores and compare them with clinicopathological parameters. The H-scores of MAOA (T; *p* = 0.005) and MAOB (T; *p* = 0.006) in tumor cells (T) were high in ACN, whereas LOX (T, S; *p* < 0.001) in tumor and stromal cells (S) and AOC3 (T; *p* < 0.001) were higher in PCC. In stromal cells, MAOA (S; *p* < 0.001) and AOC3 (S; *p* = 0.010) were more expressed in ACA than in ACC. MAOB (S) in PCC showed higher H-scores when the grading of adrenal pheochromocytoma and paraganglioma (GAPP) score was 3 or higher (*p* = 0.027). In the univariate analysis, low MAOA expression in stromal cells of ACN was associated with shorter overall survival (*p* = 0.008). In conclusion, monoamine oxidase proteins revealed differences in expression between ACN and PCC and also between benign and malignant cells.

## 1. Introduction

Adrenal gland neoplasm typically consists of an adrenal cortical tumor that originates in the cortex and pheochromocytoma (PCC) that originates in the medulla. Adrenal cortical neoplasm (ACN) is a relatively not uncommon tumor and consists of adrenal cortical adenoma (ACA) and adrenal cortical carcinoma (ACC). ACC is usually characterized by invasive growth, necrosis, and a high proliferation rate; however, low-grade ACC confined to the adrenal gland can be challenged to differentiate from ACA. Therefore, the current WHO classification of endocrine tumors [1] endorses the application of multiparameter scoring systems, including the Weiss criteria [2,3], modified Weiss criteria [4], Helsinki criteria [5], and reticulin algorithm [6] for adults, and the Lin–Weiss–Bisceglia criteria [7] for oncocytic ACN and Wieneke/AFIP [8] criteria for pediatric cases. ACC is rare and most have a dismal prognosis, and its tumorigenesis has been tremendously elucidated over the past decade, yet effective target therapy is currently absent [9]. All pheochromocytomas are considered malignant due to the lifelong risk of metastasis, and the previously used distinction between benign and malignant is no longer recommended [1]. However, there are currently no histological findings or biomarkers that can reliably predict the risk of metastasis. Various multiparameter scoring systems for risk stratification were proposed, including pheochromocytoma of the adrenal gland scaled score (PASS) [10], grading system for adrenal pheochromocytoma and paraganglioma (GAPP) [11], modified GAPP [12], and composite pheochromocytoma/paraganglioma prognostic score (COPPS) [13]. While a high proliferation index, high cellularity, necrosis, and diffuse growth pattern are encompassed as important parameters, no single system is validated for routine use.

Amine oxidase converts alkylamines into aldehydes and ammonia through oxidative cleavage. Based on the cofactor utilized, amine oxidase can be categorized into two groups: lysyl oxidase (LOX), primary amine oxidase (AOC2 and AOC3), and diamine oxidase (AOC1) are copper-dependent amine oxidases, while monoamine oxidase A (MAOA) and monoamine oxidase B (MAOB) employ flavin as a cofactor [14]. Amine oxidase is involved in cell differentiation and growth, wound healing, detoxification, and cell signaling through various metabolic pathways [15]. AOC1, notably, is an important enzyme involved in histamine degradation [16]. Its main functions are cell proliferation, allergy and inflammatory responses, and association with ischemia [17]. AOC2 is a retina-specific amine oxidase [18], while AOC3 is also known as vascular adhesion protein-1 and functions as a cell surface receptor with diverse functions [19]. In addition to its enzymatic activity as an amine oxidase, AOC3 is involved in leukocyte adhesion and inflammatory processes as an adhesion protein, participating in leukocyte trafficking [20]. LOX is an extracellular copper-dependent enzyme that oxidizes lysine residues in collagen and elastin precursors, forming aldehydes and inducing the cross-linking of collagen and elastin, which is crucial for the stability of collagen and elastin tissues [21]. MAOs are enzymes connected to the outer membrane of the mitochondria in cells composing neural tissues, such as neurons and glial cells, through a C-terminal transmembrane polypeptide fragment [22]. They play an essential role in the normal functioning of synaptic junctions by participating in the oxidative deamination of various monoamines, which are crucial for the metabolism of neurotransmitters [23].

Amine oxidase also plays a role in the biology of various cancers. LOX acts as an important mechanism for the dissemination of colorectal cancer to bone marrow [24], and affects cell proliferation, migration, invasion, and metastasis in various tumors [25]. AOC1 was reported to be associated with tumor progression in colorectal cancer [26], stomach cancer [27], and hepatocellular carcinoma [28]. In addition, AOC3 is expressed in various cancers and was reported to be associated with their prognoses [29,30,31]. The alteration of MAOA expression was related to poor prognosis in lung cancer and gastric cancer [32,33], and an association with prostate tumorigenesis and metastasis was also described [34]. MAOB is known to be highly expressed in glioma [35] and was reported to be linked with an adverse prognosis in colorectal cancer [36].

To date, few studies have been conducted on the amine oxidase family proteins in adrenal gland neoplasm. Therefore, the objective of our study was to examine the expression of amine oxidase family proteins and explore their involvement in adrenal gland neoplasm.

## 2. Materials and Methods

### 2.1. Patient Selection

Patients who underwent surgical procedures for ACN or PCC at Severance Hospital in Seoul, Republic of Korea, between January 2000 and December 2013 were included in this study. Patients who had received chemotherapy prior to surgery were excluded. The Institutional Review Board of Yonsei University Severance Hospital granted approval for this study. An endocrine pathologist (J. S. Koo) reviewed all cases. Clinicopathologic data were obtained from the patients’ medical records.

### 2.2. Tissue Microarray

Representative regions were identified on hematoxylin–eosin-stained slides and a corresponding marker was placed on the surface of the corresponding paraffin block. Five-millimeter cores were obtained from the selected regions and transferred to a recipient block arranged in a 5 × 4 grid. To mitigate any extraction bias, more than two tissue cores were sampled from each case. Each tissue core was assigned a distinct location number within the tissue microarray, which was connected to a database housing additional clinicopathological information.

### 2.3. Immunohistochemistry

The antibodies MAOA, MAOB, LOX, and AOC3 used for the immunohistochemistry (IHC) are listed in Supplementary Table S1. All IHC staining was conducted on formalin-fixed, paraffin-embedded (FFPE) tissue sections using an automated IHC staining device (Benchmark XT, Ventana Medical System, Tucson, AZ, USA). Five-micrometer-thick FFPE sections were mounted on slides and dried at 62 °C for 30 min. Heat epitope retrieval was undertaken with ethylenediaminetetraacetic acid (EDTA), pH 8.0, for 30 min in the autostainer. Following incubation with primary antibodies, the sections were subsequently incubated with biotinylated anti-mouse immunoglobulins, peroxidase-labeled streptavidin (LSAB kit, DakoCytomation, Glostrup, Denmark), and 3,3′-diaminobenzidine. Negative control samples were processed without the primary antibody, while positive control tissue was included based on the manufacturer’s recommendation. Optimal incubation times and concentrations of primary antibodies were determined via serial dilution for each immunohistochemical assay using a tissue block that was fixed and embedded in the same manner as the experimental samples.

### 2.4. Interpretation of Immunohistochemical Staining

MAOA and MAOB exhibited a cytoplasmic staining pattern, while LOX primarily displayed nuclear staining with weak cytoplasmic staining. AOC3 showed cytoplasmic and/or membrane staining patterns. The expression levels of these immunohistochemical markers were evaluated using the semi-quantitative H-score method. The scoring was performed separately for tumor cells and stromal cells (including endothelial cells, fibroblasts, and other supporting cells). The H-score ranges from 0 to 300 and is calculated by multiplying the dominant staining intensity score (0: no staining, 1: weak or barely detectable staining, 2: distinct brown staining, 3: strong dark brown staining) by the percentage (0–100%) of positive cells [37]. If the H-score exceeded the mean value, it was classified as high expression; otherwise, it was classified as low expression.

### 2.5. Statistical Analysis

To assess the statistical significance, Student’s *t*-test and Fisher’s exact test were employed. A corrected *p*-value was calculated by applying the Bonferroni multiple comparison procedure. We utilized the Kaplan–Meier method to assess survival rates, while log-rank statistics were employed to analyze disease-free survival (DFS) and overall survival (OS). DFS was measured from the date of surgery until tumor recurrence or the last follow-up visit, while OS was measured from the date of surgery until death or the last follow-up visit. Multivariate analysis was conducted using the Cox proportional hazards model, and hazard ratios (HRs) with a 95% confidence interval (CI) were reported. A two-sided *p*-value less than 0.05 was considered statistically significant. Data analysis was performed using IBM SPSS v22.00 software for Windows (IBM Corp., New York, NY, USA).

## 3. Results

### 3.1. Clinicopathologic Characteristics

General clinicopathologic characteristics of the patients with ACN and PCC are described in Supplementary Tables S2 and S3, respectively. ACC showed a larger tumor size, higher Fuhrman grade, higher mitotic rate, atypical mitosis, lower clear cell proportion, diffuse architecture, necrosis, venous/sinusoidal invasion, and capsular invasion than ACA (*p* < 0.001). An ACC variant, such as a myxoid and oncocytic type, is not included in this study. Two cases of ACN with a Weiss score of 4 or less were diagnosed as ACC, as distant metastases were present at the time of diagnosis. Recurrence, distant metastasis, and patient death occurred only with ACC. The GAPP score of PCC was 0–2 (well-differentiated type) in 113 (69.3%) cases, 3–6 (moderately differentiated type) in 50 (30.7%) cases, and no case exceeded 6 points. Tumor recurrence was found in 3 (1.8%) cases, distant metastasis in 5 (3.1%) cases, and patient death in 10 (6.1%) cases.

### 3.2. Expression of Amine Oxidase Family in Adrenal Cortical Neoplasm and Pheochromocytoma

**Figure 1:**
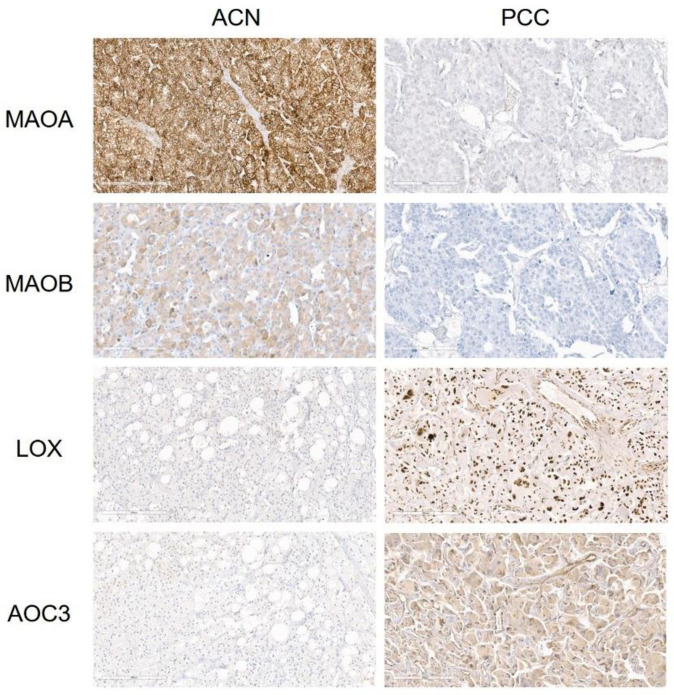
Expression of amine oxidase family in adrenal neoplasm. MAOA and MAOB in tumor cells were higher in adrenal cortical neoplasm (ACN), whereas LOX in tumor cells and stromal cells and AOC3 in tumor cells were higher in pheochromocytoma (PCC).

The H-scores of the amine oxidase family in ACN and PCC are summarized in Supplementary Table S4. The mean H-scores of ACN in the tumor cells were MAOA 180.6, LOX 163.3, AOC3 46, and MAOB 24.4, and in stromal cells, they were LOX 142.1, MAOA 126.6, AOC3 26.7, and MAOB 3.1. The mean H-scores of PCC in tumor cells were LOX 192.3, MAOA 150.8, AOC3 65.4, and MAOB 11.8, and in stromal cells, they were LOX 171.7, MAOA 132.1, AOC3 27.5, and MAOB 4.8. MAOA (T; *p* = 0.005) and MAOB (T; *p* = 0.006) in tumor cells were higher in ACN, whereas LOX was higher in tumor cells (T; *p* < 0.001) and LOX in stromal cells (S; *p* < 0.001) and AOC3 in tumor cells (T; *p* < 0.001) were higher in PCC (Table 1 and Figure 1).

**Figure 2:**
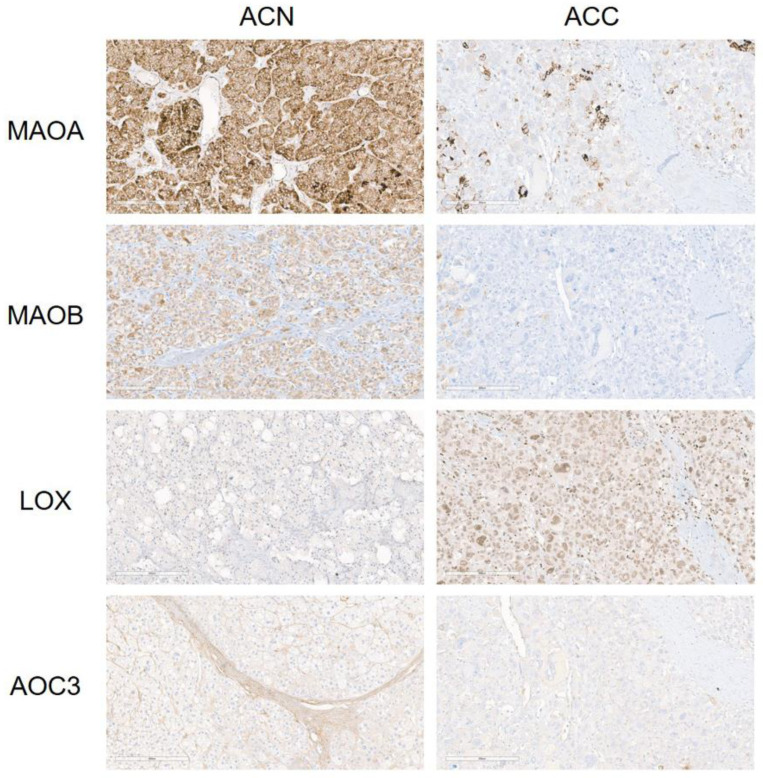
Expression of amine oxidase family in adrenal cortical neoplasm. MAOA, MAOB, and AOC3 in stromal cells showed higher expressions in adrenal cortical adenoma (ACA) than in adrenal cortical carcinoma (ACC).

The mean H-scores of ACA in tumor cells were MAOA 179.6, LOX 164, AOC3 47.3, and MAOB 25.3, and in stromal cells, they were LOX 143, MAOA 139.3, AOC3 29.4, and MAOB 3.6. The mean H-scores of ACC in tumor cells were MAOA 187.6, LOX 158.8, AOC3 37, and MAOB 18.2, and in stromal cells, they were LOX 136.7, MAOA 40.6, AOC3 7.9, and MAOB 0 (Supplementary Table S5). MAOA (S; *p* < 0.001) and AOC3 (S; *p* = 0.010) had higher H-scores in ACA than in ACC. When the H-scores were divided into low and high expressions, MAOA (S; *p* < 0.001), MAOB (S; *p* = 0.003), and AOC3 (S; *p* = 0.022) showed higher expressions in ACA than in ACC (Table 2 and Figure 2).

When comparing the H-scores according to the GAPP score in PCC, MAOB (S) showed a higher score when the GAPP score was 3 or higher (*p* = 0.027, Supplementary Table S6). Dividing the H-scores into low and high expressions, MAOB (T) showed a higher expression rate in PCC with a GAPP score of 3 or greater (*p* = 0.033, Table 3).

**Figure 3:**
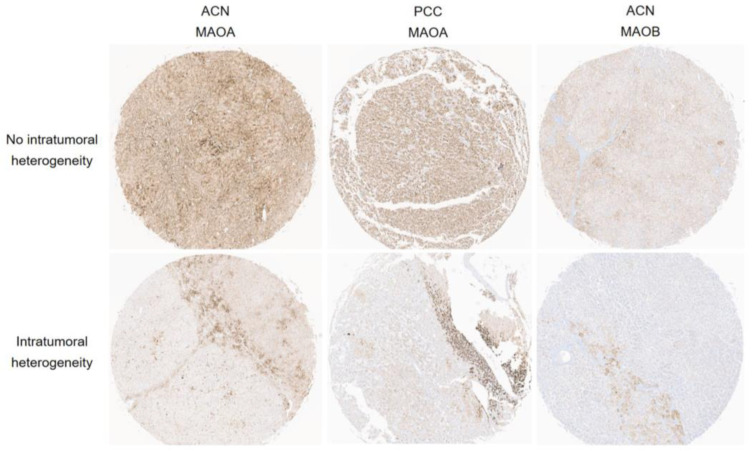
Intratumoral heterogeneity in MAOA and MAOB immunohistochemical stain for the adrenal tumor. While the majority of tumor regions show the expressions of MAOA and MAOB without exhibiting intratumoral heterogeneity, there are some cases where only certain portions of the tumor area demonstrate expression, indicating evidence of intratumoral heterogeneity. ACN, adrenal cortical neoplasm, PCC, pheochromocytoma.

In both ACN and PCC, the expression of the amine oxidase family showed a similar pattern of expression on TMA cores in most cases. In other words, there was a significant increase in the proportion of expression as the intensity of expression increased (Supplementary Table S7). However, within the amine oxidase family, MAOA and MAOB staining demonstrated evidence of intratumoral heterogeneity, with expression observed in only certain parts of the tumor area in minor cases (Figure 3).

### 3.3. Relationship between Expression of Amine Oxidase Family and Pathologic Factors of Adrenal Neoplasm

**Figure 4:**
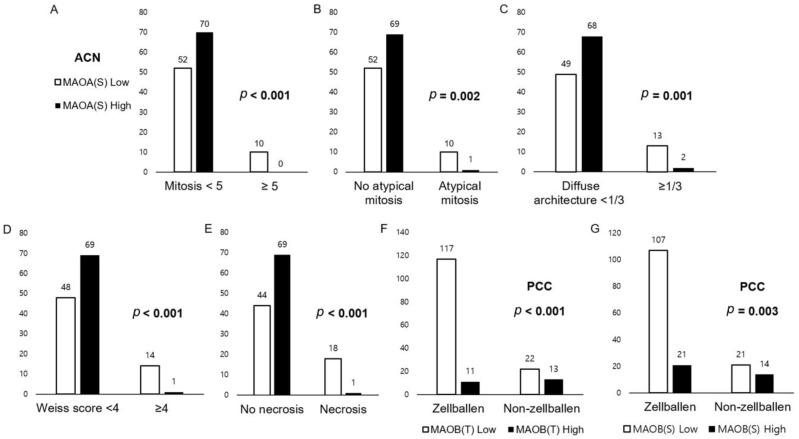
Relationship between the expression of the amine oxidase family and pathologic factors of adrenal neoplasm. In adrenal cortical neoplasm (ACN), with a higher expression of MAOA in stromal cells, lower mitosis (**A**), no atypical mitosis (**B**), lower diffuse architectural proportion (**C**), Weiss score < 4 (**D**), and no necrosis (**E**) were found. In pheochromocytoma (PCC), the zellballen pattern was associated with lower MAOB in tumor cells (**F**) and stromal cells (**G**).

In ACN, with a higher expression of MAOA (S), lower mitosis (*p* < 0.001), no atypical mitosis (*p* = 0.002), lower diffuse architectural proportion (*p* = 0.001), Weiss score < 4 (*p* < 0.001), and no necrosis (*p* < 0.001, Figure 4) were found. In PCC, the presence of a zellballen pattern was linked to decreased levels of MAOB (T; *p* < 0.001) and MAOB (S; *p* = 0.003, Figure 4).

### 3.4. The Influence of Amine Oxidase Family Expression on the Prognosis of Adrenal Cortical Neoplasm and Pheochromocytoma

**Figure 5:**
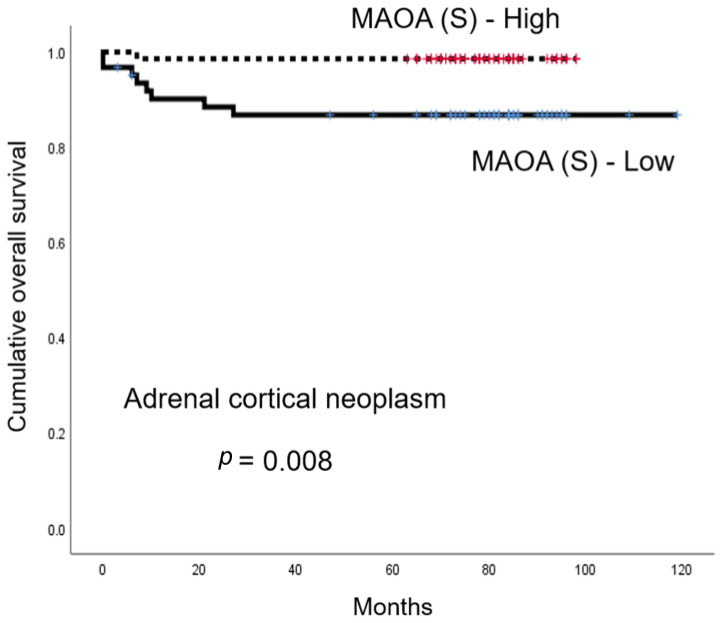
The influence of monoamine oxidase protein A in adrenal cortical neoplasm. Low MAOA expression in stromal cells of adrenal cortical neoplasm was associated with shorter overall survival.

In a univariate analysis that examined the impact of amine oxidase family expression in adrenal neoplasm on prognosis, a low MAOA (S) expression in ACN was associated with shorter OS (*p* = 0.008, Table 4 and Figure 5). There were no statistically significant factors in PCC (Table 5).

In the multivariate Cox regression analysis, diffuse architecture (proportion ≥ 1/3, hazard ratio = 12.769, 95% CI = 1.094–149, *p* = 0.042) and venous invasion (hazard ratio = 60.934, 95% CI = 4.605–806, *p* = 0.002) were the independent prognostic factors for shorter OS (Table 6).

## 4. Discussion

In the present study, amine oxidase family proteins in adrenal gland neoplasm were investigated. The expressions of MAOA (T) and MAOB (T) were high in ACN, whereas LOX (T), LOX (S), and AOC3 (T) were highly expressed in PCC. Though a direct comparison was difficult as there was no previous study comparing the status of amine oxidase family proteins between ACN and PCC, it was reported that the expressions of MAOA and MAOB are significantly reduced in PCC compared with normal adrenal medulla [38]. In normal adrenal medullas, catocholamines, such as epinephrine and norepinephrine, are inactivated by catechol-O-methyltransferase (COMT) and MAOA to metanephrine/normetanephrine (MN) and dihydroxyphenol-glycol (DHPG), respectively. On the other hand, since the expression of MAOA is significantly reduced in PCC, COMT is somewhat activated and most catocholamines are metabolized to MN [38]. Therefore, the lower expression of MAOA and MAOB in PCC compared with ACN in this study was consistent with the previous research.

There was a difference in the expressions of amine oxidase family proteins between ACA and ACC, and MAOA (S) and AOC3 (S) were higher in ACN than in ACC. ACA is usually a few stromal cells, and amine oxidase family proteins were mainly expressed in endothelial cells rather than sparse fibroblasts. It was reported that monoamine oxidase is expressed in fibroblasts [39,40,41] and endothelial cells [42], and AOC3 is expressed in endothelial cells [43,44,45] and myofibroblasts [46]. Angiogenesis-related markers showed differences in their expressions in ACA and ACC [47]. It was reported that lymphangiogenesis increased in ACA and angiogenesis increased in ACC [48]. In addition, it was observed that the endothelial area and vascular area increased in ACC compared with ACA [49]. Therefore, further research is needed to determine whether MAOA and AOC3 are expressed differently due to the difference in endothelial cell phenotype between ACA and ACC.

In PCC, the expressions of amine oxidase family proteins in stromal tissue were also mainly found in endothelial cells, and MAOB (S) revealed a higher expression when the GAPP score was 3 or higher. Angiogenesis is known to be a factor associated with malignant behavior in PCC [50,51], and since angiogenesis was activated as the GAPP score increased, it is possible that stromal MAOB expression also increased.

Low MAOA expression in stromal cells of ACN was associated with shorter OS in the present study. In previous studies, MAOA expression in tumor cells was associated with poor prognosis in several tumors [32,33,34]. Although few studies on MAOA in stromal cells have been performed, it was suggested that the expression of MAOA is increased in stromal fibroblasts of prostate cancer and that this promotes tumor cell growth through the IL-6/STAT3 signaling pathway [52]. Hence, further studies are needed on the effect of MAOA expression in the microenvironment of ACN, especially ACC, and its role as a prognostic factor.

In this study, most cases showed similar expression patterns of the amine oxidase family in tumor sites, but minor cases exhibited intratumoral heterogeneity, with expression only observed in some parts of the tumor. Intratumoral heterogeneity in various biomarkers and molecular markers has been relatively well-known across different types of tumors [53,54]. Generally, intratumoral heterogeneity can manifest as spatial intratumoral heterogeneity, which varies depending on the location within the tumor, and temporal intratumoral heterogeneity, which changes over time as the tumor progresses. The reasons for such intratumoral heterogeneity can be attributed to the dynamic nature of the tumor cells themselves, specifically the intrinsic genetic instability of the tumor cells, as well as differences in the tumor microenvironment, such as variations in growth factors, nutrients, and immune responses [55]. The clinical significance of this intratumoral heterogeneity lies in the fact that treatment responses of tumors may be influenced by the presence of biomarkers exhibiting intratumoral heterogeneity. In this study, the evaluation of intratumoral heterogeneity may have limitations when using whole tissue sections, as the expression of the amine oxidase family was examined using tissue microarrays (TMAs). There are conflicting results reported in previous studies that compared biomarker expression between TMAs and whole tissue sections, with some studies showing no significant differences [56,57] and others showing significant differences [58].

Since amine oxidase family proteins are involved in tumorigenesis and progression in various tumors, treatment methods targeting them were studied. Clorgyline, which is an MAOA inhibitor, was demonstrated to have antitumor effects against prostate cancer, glioma, and Hodgkin lymphoma [59,60,61,62]. MP-MUS, which is a prodrug activated by MAOB, reduces the proliferation of glioma cells, and Danshensu, which is an MAOB inhibitor, increases the radiosensitivity of non-small cell lung cancer [63,64]. The irreversible MAO-A/B inhibitor phenelzine (Nardil^®^) is effective in decreasing serum PSA levels in patients with recurrent prostate cancer [65]. β-Aminopropionitrile (BAPN), which is an irreversible suppressor of LOX, has anti-tumor effects in breast cancer [66], cervical cancer [67], anaplastic thyroid cancer [68], and ovarian cancer [69]. Another LOX inhibitor, namely, dextran sulfate, was shown to have anticancer effects in gastric cancer [70]. Therefore, preclinical and clinical studies on the therapeutic significance of amine oxidase family protein inhibitors could be suggested in ACC and PCC as well in the future.

In summary, we confirmed that monoamine oxidase proteins not only showed differences in expression between ACN and PCC but also differed according to the degree of malignancy.

## Figures and Tables

**Table 1:** H-scores of amine oxidase proteins in adrenal neoplasm.

H-Score (Mean ± SD)	Total N = 295 (%)	Adrenal Cortical Neoplasm *n* = 132 (%)	Pheochromocytoma *n* = 163 (%)	*p*-Value
MAOA (T)	164.4 ± 90.9	180.6 ± 90.5	150.8 ± 89.3	**0.005**
MAOA (S)	129.7 ± 79.4	126.7 ± 80.8	132.2 ± 78.5	0.555
MAOB (T)	17.4 ± 39.3	24.4 ± 40.7	11.8 ± 37.4	**0.006**
MAOB (S)	4.0 ± 19.6	3.1 ± 7.1	4.8 ± 25.6	0.469
LOX (T)	179.4 ± 67.3	163.3 ± 66.1	192.3 ± 65.6	**<0.001**
LOX (S)	158.5 ± 68.7	142.1 ± 62.8	171.8 ± 70.6	**<0.001**
AOC3 (T)	56.7 ± 44.5	46.1 ± 47.9	65.4 ± 39.7	**<0.001**
AOC3 (S)	27.2 ± 29.0	26.7 ± 32.4	27.5 ± 26.0	0.798

S, stromal cell; SD, standard deviation; T, tumor cell. Values in bold indicate statistically significant results.

**Table 2:** Expressions of amine oxidase proteins in adrenal cortical neoplasm.

Parameters	Total N = 132 (%)	Adrenal Cortical Adenoma, *n* = 115 (%)	Adrenal Cortical Carcinoma, *n* = 17 (%)	*p*-Value
MAOA (T)				0.606
Low	54 (40.9)	46 (40.0)	8 (47.1)	
High	78 (59.1)	69 (60.0)	9 (52.9)	
MAOA (S)				**<0.001**
Low	62 (47.0)	46 (40.0)	16 (94.1)	
High	70 (53.0)	69 (60.0)	1 (5.9)	
MAOB (T)				0.341
Low	105 (79.5)	93 (80.9)	12 (70.6)	
High	27 (20.5)	22 (19.1)	5 (29.4)	
MAOB (S)				**0.003**
Low	93 (70.5)	76 (66.1)	17 (100.0)	
High	39 (29.5)	39 (33.9)	0 (0.0)	
LOX (T)				0.788
Low	48 (36.4)	41 (35.7)	7 (41.2)	
High	84 (63.6)	74 (64.3)	10 (58.8)	
LOX (S)				0.794
Low	56 (42.4)	48 (41.7)	8 (47.1)	
High	76 (57.6)	67 (58.3)	9 (52.9)	
AOC3 (T)				0.196
Low	72 (54.5)	60 (52.2)	12 (70.6)	
High	60 (45.5)	55 (47.8)	5 (29.4)	
AOC3 (S)				**0.022**
Low	93 (70.5)	77 (67.0)	16 (94.1)	
High	39 (29.5)	38 (33.0)	1 (5.9)	

S, stromal cell; T, tumor cell. Values in bold indicate statistically significant results.

**Table 3:** Expression of amine oxidase family in pheochromocytoma according to GAPP score.

Parameters	Total N = 163 (%)	GAPP < 3 *n* = 113 (%)	GAPP ≥ 3 *n* = 50 (%)	*p*-Value
MAOA (T)				0.610
Low	67 (41.1)	48 (42.5)	19 (38.0)	
High	96 (58.9)	65 (57.5)	31 (62.0)	
MAOA (S)				0.235
Low	81 (49.7)	60 (53.1)	21 (42.0)	
High	82 (50.3)	53 (46.9)	29 (58.0)	
MAOB (T)				**0.033**
Low	139 (85.3)	101 (89.4)	38 (76.0)	
High	24 (14.7)	12 (10.6)	12 (24.0)	
MAOB (S)				0.215
Low	128 (78.5)	92 (81.4)	36 (72.0)	
High	35 (21.5)	21 (18.6)	14 (28.0)	
LOX (T)				0.728
Low	63 (38.7)	45 (39.8)	18 (36.0)	
High	100 (61.3)	68 (60.2)	32 (64.0)	
LOX (S)				0.732
Low	70 (42.9)	50 (44.2)	20 (40.0)	
High	93 (57.1)	63 (55.8)	30 (60.0)	
AOC3 (T)				0.864
Low	68 (41.7)	48 (42.5)	20 (40.0)	
High	95 (58.3)	65 (57.5)	65 (57.5)	
AOC3 (S)				0.731
Low	97 (59.5)	66 (58.4)	31 (62.0)	
High	66 (40.5)	47 (41.6)	19 (38.0)	

GAPP, grading system for adrenal pheochromocytoma and paraganglioma; S, stromal cell; SD, standard deviation; T, tumor cell. Values in bold indicate statistically significant results.

**Table 4:** Univariate analysis of the effect of amine oxidase family expression in adrenal cortical neoplasm on survival.

Parameter	No. of Patients /Recurrence /Death	Disease-Free Survival		Overall Survival	
		Mean Survival Months (95% CI)	*p*-Value	Mean Survival Months (95% CI)	*p*-Value
MAOA (T)			0.839		0.096
Low	54/1/6	107 (103–110)		97 (89–106)	
High	78/2/3	116 (112–120)		114 (110–119)	
MAOA (S)			-		**0.008**
Low	62/3/8	-		104 (95–113)	
High	70/0/1	-		96 (94–99)	
MAOB (T)			-		0.850
Low	105/3/7	-		111 (106–116)	
High	27/0/2	-		101 (90–111)	
MAOB (S)			-		-
Low	93/3/9	-		-	
High	39/0/0	-		-	
LOX (T)			0.306		0.345
Low	48/3/2	105 (99–110)		105 (99–110)	
High	84/1/7	117 (115–120)		109 (102–116)	
LOX (S)			0.423		0.535
Low	56/2/3	105 (100–110)		103 (98–109)	
High	76/1/6	117 (114–120)		110 (103–116)	
AOC3 (T)			-		0.988
Low	72/3/5	-		111 (105–117)	
High	60/0/4	-		91 (85–97)	
AOC3 (S)			-		-
Low	93/3/9	-		-	
High	39/0/0	-		-	

CI, confidence interval; S, stromal cell; T, tumor cell. Values in bold indicate statistically significant results.

**Table 5:** Univariate analysis of the effect of amine oxidase family expression in pheochromocytoma on survival.

Parameter	No. of Patients /Recurrence /Death	Disease-Free Survival		Overall Survival	
		Mean Survival Months (95% CI)	*p*-Value	Mean Survival Months (95% CI)	*p*-Value
MAOA (T)			-		0.094
Low	67/0/7	-		113 (102–124)	
High	96/3/3	-		160 (153–167)	
MAOA (S)			0.730		0.829
Low	81/1/5	138 (134–143)		128 (118–138)	
High	82/2/5	154 (145–163)		152 (140–165)	
MAOB (T)			-		-
Low	139/3/10	-		-	
High	24/0/0	-		-	
MAOB (S)			-		-
Low	128/3/10	-		-	
High	35/0/0	-		-	
LOX (T)			0.313		0.157
Low	63/2/6	154 (146–163)		148 (134–162)	
High	100/1/4	151 (144–159)		154 (142–165)	
LOX (S)			0.349		0.217
Low	70/2/6	154 (146–163)		148 (134–162)	
High	93/1/4	151 (143–159)		153 (142–165)	
AOC3 (T)			0.770		0.908
Low	68/1/4	157 (151–163)		149 (138–160)	
High	95/2/6	151 (141–160)		149 (136–163)	
AOC3 (S)			0.195		0.099
Low	97/1/9	159 (155–162)		142 (130–153)	
High	66/2/1	143 (121–164)		163 (156–170)	

CI, confidence interval; S, stromal cell; T, tumor cell.

**Table 6:** Multivariate analysis of the overall survival of patients with adrenal cortical neoplasm.

Parameter	Hazard Ratio	95% CI	*p*-Value
Fuhrman grade			0.797
1 or 2 versus 3 or 4	0.654	0.026–16.63	
Mitosis (/50 HPFs)			0.393
≤5 versus >5	3.315	0.212–51.76	
Atypical mitosis			0.362
Absent versus present	2.589	0.335–19.99	
Clear cell proportion			0.240
≥25% versus <25%	5.684	0.313–103	
Diffuse architecture (proportion)			**0.042**
<1/3 versus ≥1/3	12.796	1.094–149	
Venous invasion			**0.002**
Absent versus present	60.934	4.605–806	
Capsular invasion			0.965
Absent versus present	0.954	0.115–7.885	
Weiss score			0.839
<4 versus ≥4	0.680	0.017–27.86	
MAOA (S) expression			0.423
Low versus high	0.215	0.005–9.273	

CI, confidence interval; S, stromal cell. Values in bold indicate statistically significant results.

## Data Availability

All study data are included in the article and Supplementary Materials.

## Supplementary Materials

The following supporting information can be downloaded from https://www.mdpi.com/article/10.3390/biomedicines11071896/s1: Table S1. Source, clone, and dilution of antibodies; Table S2. Basal characteristics of adrenal cortical neoplasm; Table S3. Basal characteristics of pheochromocytoma; Table S4. H-scores of monoamine oxidase proteins in adrenal neoplasm; Table S5. H-scores of monoamine oxidase proteins in adrenal cortical neoplasm; Table S6. H-scores of monoamine oxidase proteins in pheochromocytoma according to GAPP score; Table S7. Difference in IHC proportion score based on the IHC intensity score in adrenal neoplasm.
