# Supplementary material for: Expression of Amine Oxidase Proteins in Adrenal Cortical Neoplasm and Pheochromocytoma
Source: Biomedicines. 2023 Jul 4;11(7):1896. doi: 10.3390/biomedicines11071896 (PMC10376964; doi:10.3390/biomedicines11071896)
Supplement: Supplementary file 1 [file biomedicines-11-01896-s001.zip › biomedicines-2469358-supplementary.pdf]

Supplementary Materials

Table S1. Source, clone, and dilution of antibodies.

Antibody	Company	Clone	Dilution
Monoamine oxidase A	Abcam, Cambridge, UK	EPR7101	1:100
Monoamine oxidase B	Abcam, Cambridge, UK	Polyclonal	1:100
Lysyl oxidase (LOX)	Abcam, Cambridge, UK	Polyclonal	1:100
Amine oxidase (AOC3)	Abcam, Cambridge, UK	Polyclonal	1:1000

Table S2. Basal characteristics of adrenal cortical neoplasm.

Parameters	Total N = 132 (%)	Adrenal Cortical Adenoma *n* = 115 (%)	Adrenal Cortical Carcinoma *n* = 17 (%)	p-Value
Age (year, mean ± SD)	47.5 ± 145	48.4 ± 12.2	41.0 ± 25.1	**0.048**
Sex				0.107
Male	40 (30.3)	32 (27.8)	8 (47.1)	
Female	92 (69.7)	83 (72.2)	9 (52.9)	
Tumor size (cm, mean ± SD)	3.6 ± 3.7	2.5 ± 1.3	10.9 ± 5.8	**<0.001**
Fuhrman grade				**<0.001**
1 and 2	106 (80.3)	102 (88.7)	4 (23.5)	
3 and 4	26 (19.7)	13 (11.3)	13 (76.5)	
Mitosis				**<0.001**
≤ 5/50 HPFs	122 (92.4)	115 (100.0)	7 (41.2)	
> 5/50 HPFs	10 (7.6)	0 (0.0)	10 (58.8)	
Atypical mitosis				**<0.001**
Absent	121 (91.7)	114 (99.1)	7 (41.2)	
Present	11 (8.3)	1 (0.9)	10 (58.8)	
Clear cell proportion				**<0.001**
≥ 25%	96 (72.7)	95 (82.6)	1 (5.9)	
< 25%	36 (27.3)	20 (17.4)	16 (94.1)	
Diffuse architecture				**<0.001**
< 1/3	117 (88.6)	111 (96.5)	6 (35.3)	
≥ 1/3	15 (11.4)	4 (3.5)	11 (64.7)	
Necrosis				**<0.001**
Absent	113 (85.6)	113 (98.3)	0 (0.0)	
Present	19 (14.4)	2 (1.7)	17 (100.0)	
Venous invasion				**<0.001**
Absent	126 (95.5)	115 (100.0)	11 (64.7)	
Present	6 (4.5)	0 (0.0)	6 (35.3)	
Sinusoidal invasion				**<0.001**
Absent	126 (95.5)	115 (100.0)	11 (64.7)	
Present	6 (4.5)	0 (0.0)	6 (35.3)	
Capsular invasion				**<0.001**
Absent	117 (88.6)	111 (96.5)	6 (35.3)	
Present	15 (11.4)	4 (3.5)	11 (64.7)	
Weiss total score				**<0.001**
< 4	117 (88.6)	115 (100.0)	2 (11.8) *	
≥ 4	15 (11.4)	0 (0.0)	15 (88.2)	
Recurrence	3 (2.3)	0 (0.0)	3 (17.6)	**<0.001**
Distant metastasis	7 (5.3)	0 (0.0)	7 (41.2)	**<0.001**
Patient death	9 (6.8)	0 (0.0)	9 (52.9)	**<0.001**

SD, standard deviation; * Although the Weiss score was 4 or less, it was diagnosed as adrenal cortical carcinoma as metastases were present at the time of diagnosis. Values in bold indicate statistically significant results.

Table S3. Basal characteristics of pheochromocytoma.

Parameters	Total, N = 163 (%)
Age (year, mean ± SD)	48.2 ± 14.3
Sex	
Male	65 (39.9)
Female	98 (60.1)
Tumor size (cm, mean ± SD)	3.3 ± 5.0
Histologic pattern	
Zellballen	128 (78.5)
Non-Zellballen	35 (21.5)
Cellularity	
Low	24 (14.7)
Moderate	119 (73.0)
High	20 (12.3)
Comedo necrosis	
Absent	162 (99.4)
Present	1 (0.6)
Vascular or capsular invasion	
Absent	107 (65.6)
Present	56 (34.4)
Ki–67 labeling index (%)	
< 1	123 (75.5)
1–3	31 (19.0)
> 3	9 (5.5)
Catecholamine type	
Non-norepinephrine type	129 (79.1)
Norepinephrine type	34 (20.9)
GAPP score	
0–2 (well-differentiated type)	113 (69.3)
3–6 (moderately differentiated type)	50 (30.7)
7–10 (poorly differentiated type)	0 (0.0)
Tumor recurrence	3 (1.8)
Distant metastasis	5 (3.1)
Patient death	10 (6.1)

GAPP, grading system for adrenal pheochromocytoma and paraganglioma; SD, standard deviation.

Table S4. H-scores of amine oxidase proteins in adrenal neoplasm.

Parameters	Adrenal Cortical Neoplasm *n* = 132 (%)		Pheochromocytoma *n* = 163 (%)	
	H-Score (mean ± SD)	H-Score (range)	H-Score (Mean ± SD)	H-Score (range)
MAOA (T)	180.6 ± 90.5	0–300	150.8 ± 89.3	0–300
MAOA (S)	126.6 ± 80.8	5–300	132.1 ± 78.5	5–300
MAOB (T)	24.4 ± 40.7	0–200	11.8 ± 37.4	0–300
MAOB (S)	3.1 ± 7.1	0–40	4.8 ± 25.6	0–300
LOX (T)	163.3 ± 66.1	0–300	192.3 ± 65.6	60–300
LOX (S)	142.1 ± 62.7	0–300	171.7 ± 70.6	60–300
AOC3 (T)	46.0 ± 47.9	0–200	65.4 ± 39.7	0–200
AOC3 (S)	26.7 ± 32.4	0–140	27.5 ± 26.0	0–120

S, stromal cell; SD, standard deviation; T, tumor cell.

Table S5. H-scores of amine oxidase proteins in adrenal cortical neoplasm.

H-Score (Mean ± SD)	Total N = 132 (%)	Adrenal Cortical Adenoma, *n* = 115 (%)	Adrenal Cortical Carcinoma, *n* = 17 (%)	*p*-Value
MAOA (T)	180.6 ± 90.5	179.6 ± 84.4	187.6 ± 127.6	0.734
MAOA (S)	126.7 ± 80.8	139.3 ± 77.2	40.6 ± 44.9	**<0.001**
MAOB (T)	24.4 ± 40.7	25.3 ± 41.8	18.2 ± 32.6	0.504
MAOB (S)	3.1 ± 7.1	3.6 ± 7.5	0.0 ± 0.0	0.053
LOX (T)	163.3 ± 66.1	164.0 ± 60.8	158.8 ± 96.6	0.764
LOX (S)	142.1 ± 62.8	143.0 ± 58.5	136.7 ± 88.2	0.691
AOC3 (T)	46.1 ± 47.9	47.3 ± 47.2	37.0 ± 53.5	0.409
AOC3 (S)	26.7 ± 32.4	29.4 ± 33.7	7.9 ± 8.1	**0.010**

S, stromal cell; SD, standard deviation; T, tumor cell. Values in bold indicate statistically significant results.

Table S6. H-scores of amine oxidase proteins in pheochromocytoma according to GAPP score.

H-Score (Mean ± SD)	Total N = 189 (%)	GAPP < 3 *n* = 138 (%)	GAPP ≥ 3 *n* = 51 (%)	*p*-Value
MAOA (T)	150.8 ± 89.3	149.7 ± 89.2	153.1 ± 90.3	0.828
MAOA (S)	132.2 ± 78.5	126.4 ± 77.0	145.2 ± 81.0	0.160
MAOB (T)	11.8 ± 37.4	8.7 ± 26.8	18.9 ± 53.9	0.111
MAOB (S)	4.8 ± 25.6	1.8 ± 4.5	11.5 ± 45.4	**0.027**
LOX (T)	192.3 ± 65.6	190.1 ± 62.6	197.4 ± 72.4	0.519
LOX (S)	171.8 ± 70.6	168.3 ± 68.8	179.6 ± 74.6	0.349
AOC3 (T)	65.4 ± 39.7	62.0 ± 38.7	73.0 ± 41.2	0.106
AOC3 (S)	27.5 ± 26.0	28.1 ± 26.0	26.4 ± 26.1	0.703

GAPP, grading system for adrenal pheochromocytoma and paraganglioma; S, stromal cell; SD, standard deviation; T, tumor cell.

Table S7. Difference in IHC proportion score based on IHC intensity score in adrenal neoplasm.

IHC Proportion Score (mean ± SD)	Adrenal Cortical Neoplasm				Pheochromocytoma			
	IHC Intensity Score			*p*-Value	IHC Intensity Score			*p*-Value
	1	2	3		1	2	3	
MAOA (T)	64.3 ± 38.2	88.1 ± 22.6	93.8 ± 20.7	**<0.001**	62.3 ± 35.9	91.4 ± 12.0	95.3 ± 8.5	**<0.001**
MAOA (S)	32.2 ± 28.6	68.3 ± 29.5	83.8 ± 24.6	**<0.001**	53.4 ± 30.3	79.2 ± 19.5	90.0 ± 16.8	**<0.001**
MAOB (T)	23.0 ± 26.0	40.5 ± 37.1	n/a	**<0.001**	21.6 ± 21.7	65.8 ± 31.3	100.0 ± 0.0	**<0.001**
MAOB (S)	9.8 ± 8.6	20.0 ± 0.0	n/a	**<0.001**	10.1 ± 8.0	19.0 ± 23.0	100.0 ± 0.0	**<0.001**
LOX (T)	80.8 ± 14.4	91.0 ± 12.1	97.5 ± 4.5	**<0.001**	87.0 ± 11.4	95.7 ± 6.9	99.0 ± 3.0	**<0.001**
LOX (S)	70.9 ± 14.9	81.2 ± 12.1	95.5 ± 7.2	**<0.001**	72.7 ± 11.3	87.2 ± 12.0	95.1 ± 8.7	**<0.001**
AOC3 (T)	37.1 ± 28.7	77.8 ± 14.7	n/a	**<0.001**	62.2 ± 29.2	83.1 ± 29.3	n/a	**<0.001**
AOC3 (S)	20.4 ± 19.8	44.0 ± 22.6	n/a	**<0.001**	25.7 ± 23.4	36.2 ± 16.8	n/a	0.070

Values in bold indicate statistically significant results.
